# Expression and functional characterization of CD33 transcript variants in human acute myeloid leukemia

**DOI:** 10.18632/oncotarget.9674

**Published:** 2016-05-27

**Authors:** George S. Laszlo, Kimberly H. Harrington, Chelsea J. Gudgeon, Mary E. Beddoe, Matthew P. Fitzgibbon, Rhonda E. Ries, Jatinder K. Lamba, Martin W. McIntosh, Soheil Meshinchi, Roland B. Walter

**Affiliations:** ^1^ Clinical Research Division, Fred Hutchinson Cancer Research Center, Seattle, WA, USA; ^2^ Public Health Sciences Division, Fred Hutchinson Cancer Research Center, Seattle, WA, USA; ^3^ Department of Pharmacotherapy and Translational Research College of Pharmacy, University of Florida, Gainesville, FL, USA; ^4^ Children's Oncology Group, Arcadia, CA, USA; ^5^ Department of Pediatrics, University of Washington, Seattle, WA, USA; ^6^ Department of Medicine, Division of Hematology, University of Washington, Seattle, WA, USA; ^7^ Department of Epidemiology, University of Washington, Seattle, WA, USA

**Keywords:** acute myeloid leukemia, antigen, CD33, immunotherapy, splice variants

## Abstract

With the demonstration of improved survival of some acute myeloid leukemia (AML) patients with the CD33 antibody-drug conjugate, gemtuzumab ozogamicin (GO), CD33 has been validated as a target for antigen-specific immunotherapy. Since previous studies identified a CD33 splice variant missing exon 2 (CD33^∆E2^) and, consequently, the immune-dominant membrane-distal V-set domain, we investigated the expression and functional characteristics of CD33 transcript variants in AML. In primary AML specimens, we not only found full-length CD33 (CD33^FL^) and CD33^∆E2^ but also corresponding variants containing an alternate exon 7 predicted to encode a CD33 protein lacking most of the intracellular domain (CD33^E7a^ and, not previously described, CD33^∆E2,E7a^) in almost all cases. In acute leukemia cell sublines engineered to express individual CD33 splice variants, all splice variants had endocytic properties. CD33^FL^ and CD33^E7a^ mediated similar degrees of GO cytotoxicity, whereas CD33^∆E2^ and CD33^∆E2,E7a^ could not serve as target for GO. Co-expression of CD33^∆E2^ did not interfere with CD33^FL^ endocytosis and did not impact CD33^FL^-mediated GO cytotoxicity. Together, our findings document a greater-than-previously thought complexity of CD33 expression in human AML. They identify CD33 variants that lack exon 2 and are not recognized by current CD33-directed therapeutics as potential target for future unconjugated or conjugated antibodies.

## INTRODUCTION

CD33, a member of the sialic acid-binding immunoglobulin (Ig)-like lectin (Siglec) family, is a 67 kD single pass transmembrane glycoprotein with endocytic properties that is primarily expressed on normal multipotent myeloid precursors, unipotent colony-forming cells, and maturing granulocytes and monocytes [1]. CD33 is comprised of an amino-terminal variable (V)-set Ig-like domain mediating sialic acid binding followed by a C2-set Ig-like domain in its extracellular region, a transmembrane domain, and a cytoplasmic tail that contains 2 conserved tyrosine-based inhibitory signaling motifs. Upon phosphorylation, the latter provide docking sites for the recruitment and activation of the Src homology-2 (SH2) domain-containing tyrosine phosphatases SHP-1 and SHP-2 [1]. Increasing evidence suggests that CD33 and related Siglecs with inhibitory signaling motifs modulate inflammatory and immune responses through dampening of tyrosine kinase-driven signaling pathways [2]. For CD33, the precise physiological functions have remained unclear, but recently, genome-wide association studies have identified variants of CD33 as a major risk factor for Alzheimer's disease, suggesting an important role in neurodegeneration [2].

Restricted expression patterns render Siglecs appealing targets for antibody- and glycan-based therapeutics [3]. Consistent with its characteristic as a myeloid differentiation antigen, CD33 is expressed on at least a subset of malignant blasts in nearly all patients with acute myeloid leukemia (AML) and also, perhaps, in underlying leukemia stem cells in some cases [1, 4]. There has therefore been a long-standing interest in the development of CD33-directed unconjugated and conjugated monoclonal antibodies for the treatment of AML. To date, gemtuzumab ozogamicin (GO), a humanized CD33 antibody conjugated to a calicheamicin-γ_1_ derivative, has had the most success in the clinic. Several randomized trials have demonstrated that the addition of GO to induction chemotherapy reduces the relapse risk and improves survival of adults with newly diagnosed AML and favorable- or intermediate-risk features [5]. These studies are complemented by a large Children's Oncology Group (COG) trial of > 1,000 pediatric patients in which GO reduced the relapse risk and improved event-free survival irrespective of the cytogenetic disease risk [6]. Although GO was withdrawn from the commercial market in most countries in 2010, these results have validated CD33 as therapeutic target in AML and have sparked renewed interest in CD33-directed immunotherapies [1].

UCSC genome browser and Ensembl databases contain 3 unique, validated CD33 transcript variants supported by at least one non-suspect mRNA sequence, NM_001772, NM_001082618 and NM_001177608. NM_001772 represents full-length CD33 (CD33^FL^) consisting of 7 coding exons and a 364 amino acid protein [7]. NM_001082618 skips exon 2 resulting in CD33 with a deleted exon 2 (CD33^∆E2^) and a 237 amino acid protein [8–11]. NM_001177608 uses an alternate exon 7, exon 7a (CD33^E7a^) [10], which is predicted to encode an early translational stop, resulting in a 310 amino acid protein with truncation of the 54 c-terminal amino acids of the intracellular domain relative to CD33^FL^. The existence of CD33 variants lacking exon 2 is relevant for therapeutic targeting of CD33 since the V-set domain, which is encoded by exon 2, contains immune-dominant epitope(s) [8, 12] recognized by all currently clinically exploited CD33-directed therapeutics. Moreover, our previous studies in cell lines engineered to express mutant forms of CD33 demonstrated that CD33 endocytosis is largely limited and determined by the intracellular domain of CD33 and that changes in this domain significantly impact CD33 internalization [13–15]. It is therefore conceivable that transcript variants that lack most of the intracellular domain of CD33 differ in their endocytic properties from the wild-type protein. Here, we investigated to what degree CD33 transcript variants are found in AML, what the endocytic properties are of these variants, and whether their co-expression affects the ability of CD33^FL^ to serve as a target for therapeutic antibodies.

## RESULTS

### Expression of CD33 splice variants in primary AML specimens

**Figure 1:**
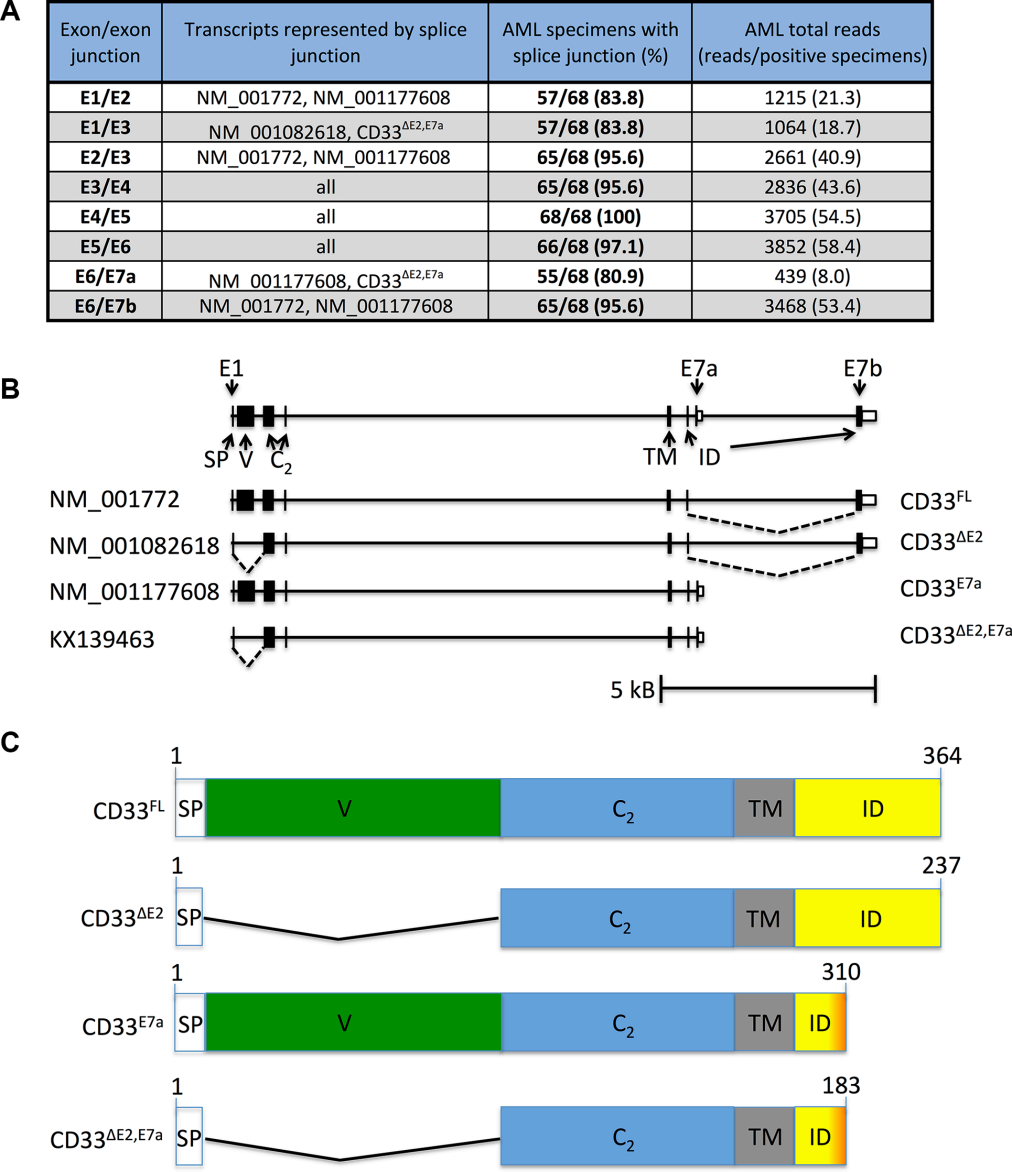
RNA sequencing to identify CD33 splice variants in human AML (**A**) Summary of RNAseq results from 68 AML specimens, with provision of CD33 exon/exon boundaries (column 1) and known transcripts containing these specific exon/exon boundaries (column 2). Also indicated is the number of specimens with a positive read for each junction (columns 3) as well as total reads (columns 4). (**B**) Genomic structure of CD33^FL^ and CD33 splice variants. Located at chromosome 19q13.41, CD33^FL^ encodes an amino-terminus signal peptide (SP) of 16 AA (exon 1), a sialic acid-binding amino-terminal V-set Ig-like domain (V, exon 2), a C_2_-set Ig-like domain (C_2_, exon 3 and 4), a transmembrane domain (TM, exon 5), and an intracellular domain (ID, exon 6 and 7a or 7b). Besides CD33^FL^, RNAseq suggested our studies identified 3 CD33 mRNA transcript variants, including two reported splice variants, CD33^∆E2^ and CD33^E7a^, and a previously unknown variant, CD33^∆E2,E7a^. Skipped exons (dotted lines) and approximate location of variant specific primers are indicated. (**C**) Functional domains of CD33^FL^ and CD33 splice variants. CD33^∆E2^ lacks 124 amino acids encoded by exon 2 and, as a result, the V-set domain, while CD33^E7a^ lacks 54 carboxy-terminal amino acids due to an early translation stop signal residing in exon 7a. CD33^∆E2,E7a^ lacks both exon 2 and the 54 carboxy-terminal amino acids. Exon/exon boundaries were confirmed through gel extraction of PCR products shown, subsequent purification, and sequence analysis.

To survey the pattern of CD33 splice variant expression in human AML, we took advantage of whole transcriptome RNA sequencing (RNAseq) data from 61 pre-treatment bone marrow and 7 peripheral blood specimens collected from pediatric patients with newly diagnosed AML. CD33 coding sequence splice junctions were filtered for junctions with 10% prevalence at a threshold of ≥ 10 reads per specimen, resulting in 8 observed splice junctions (E1/E2, E1/E3, E2/E3, E3/E4, E4/E5, E5/E6, E6/E7a, E6/E7b; Figure 1A). These 8 splice junctions were consistent with the 3 known and well-supported protein coding CD33 transcripts (NM_001772, NM_001082618, and NM_001177608; Figure 1B). Of the 8 junctions, 6 were shared by 2 or more known CD33 variants, whereas 2 were unique to one of two variants, NM_001082618 (E1/E3; specific for CD33^∆E2^) and NM_001177608 (E6/E7a; specific for CD33^E7a^). The latter 2 were found in 57/68 (85.3%) and 55/68 (80.9%) of the specimens (Figure 1C). The observed CD33 splice junctions in our RNAseq dataset also offered the possibility of a previously unknown fourth, distinct CD33 transcript that lacked exon 2 and used exon E7a instead of E7b (CD33^∆E2,E7a^).

**Figure 2:**
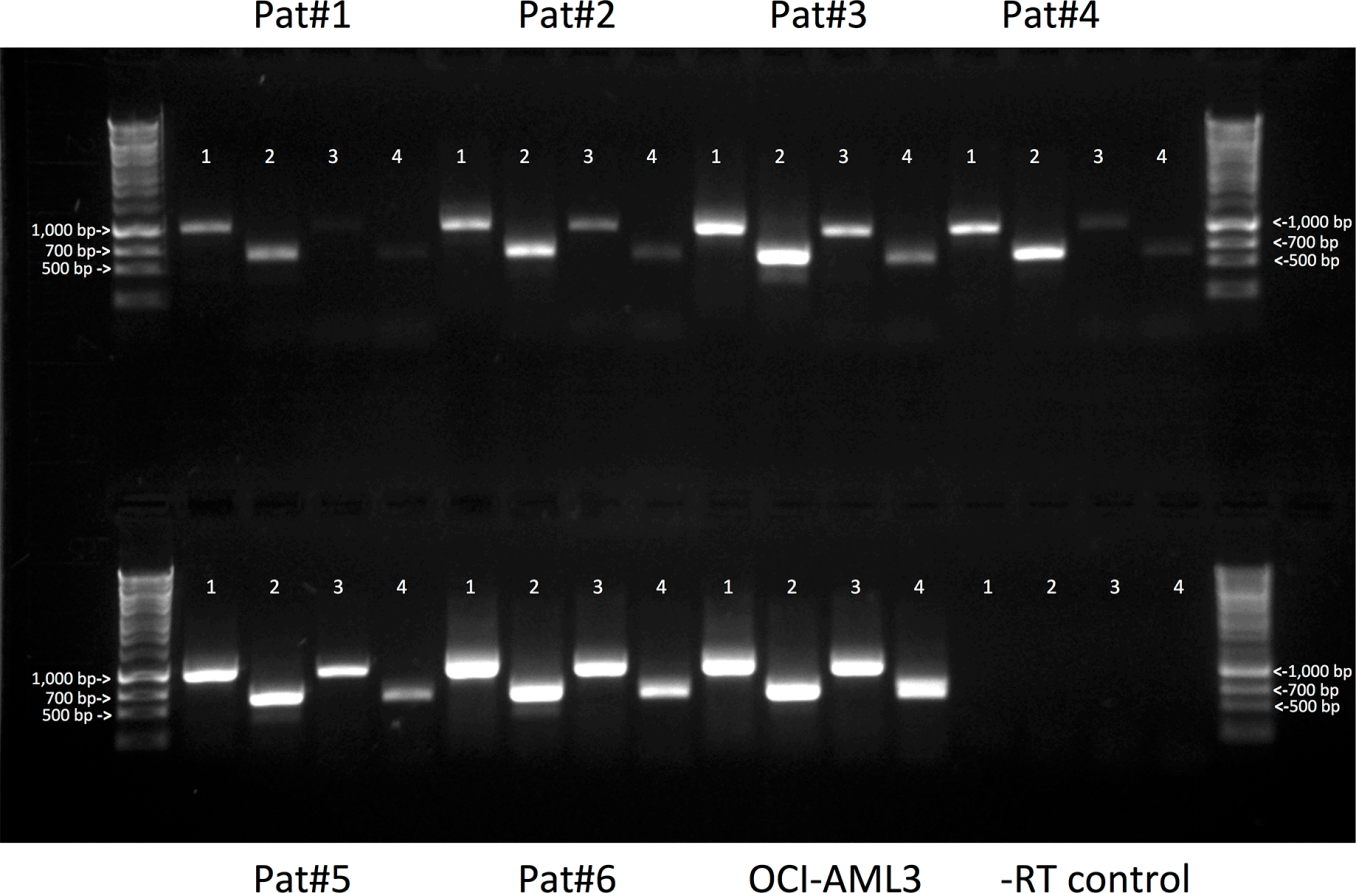
Expression of CD33 splice variants in primary AML specimens Total RNA was isolated from 29 primary AML specimens and assayed for expression of mRNA for CD33^FL^, CD33^∆E2^, CD33^E7a^, and CD33^∆E2,E7a^, using variant-specific primers. Amplicons were electrophoretically separated in 0.9% agarose gel and visualized with ethidium bromide. Shown are results from 6 representative samples; also shown are a positive control (OCI-AML3 cells), a negative control (−reverse transcriptase [−RT]), as well as a sizing ladder. Lane 1: CD33^FL^ (expected amplicon size: 980 bp); lane 2: CD33^∆E2^ (expected amplicon size: 617 bp); lane 3: CD33^E7a^ (expected amplicon size: 965 bp); and lane 4: CD33^∆E2,E7a^ (expected amplicon size: 604 bp).

To further study CD33 variants in human AML and validate the findings obtained with RNAseq, we designed transcript-specific primers and used RT-PCR to assess expression of the CD33^FL^, CD33^∆E2^, CD33^E7a^, and CD33^∆E2,E7a^ transcripts in 29 pre-treatment specimens from adults with AML. Basic characteristics of these specimens are summarized in Table 1. In the majority of specimens (27/29 [93.1%]), amplicons corresponding to the expected sizes of all 4 CD33 transcripts were found, whereas in the remaining 2 specimens (6.9%; samples #14 and #21), only 3 transcripts (CD33^FL^, CD33^∆E2^, and CD33^E7a^) were detectable (Figure 2). Three unique amplicons per CD33 transcript were subjected to Sanger sequencing to confirm the lack of exon 2 in CD33^∆E2^, use of exon 7a in CD33^E7a^, and lack of exon 2 combined with use of exon 7a in CD33^∆E2,E7a^; the latter sequence was subsequently deposited in GenBank (accession no. KX139463). In 7 cases, we had bone marrow specimens (containing AML cells) and paired peripheral blood specimens (without morphologic evidence of AML cells) available that were obtained on the same day. In all cases, the pattern of CD33 isoform expression was very similar in the peripheral blood and the marrow specimen, i.e. all CD33 isoforms could be identified in non-blast cells.

**Table 1:** Basic characteristics of primary AML specimens used to study expression of CD33 splice variants by RT-PCR

Sample #	Age	Disease stage	Karyotype	Cytogenetic risk	FLT3/ITD	NPM1	Source	Blasts (%)
1	68.90	Refractory	Hypodiploid with monosomy of chromosome 5, 11, 13, and 17	Adverse	Neg	Neg	PB	86.9
2	67.35	Relapse	Normal	Intermediate	Pos	Neg	BM	94.7
3	65.30	Relapse	Normal	Intermediate	Pos	Pos	BM	89.2
4	53.01	Diagnosis	Normal	Intermediate	Pos	Neg	PB	87.4
5	71.52	Relapse	Normal	Intermediate	Pos	Neg	BM	82.0
6	78.79	Diagnosis	Normal	Intermediate	ND	ND	PB	95.1
7	63.00	Diagnosis	46,XX,t(3;5)(q21;q22)[9]/46,XX[11]	Intermediate	Neg	Neg	PB	92.3
8	67.14	Relapse	Normal	Intermediate	Neg	Pos	BM	79.4
9	61.07	Diagnosis	Normal	Intermediate	Neg	Pos	BM	94.1
10	78.03	Diagnosis	46,XX,del(7)(q32)[2]/46,XX[19]	Adverse	Neg	Neg	BM	90.9
11	72.63	Diagnosis	47,XY,add(1),del(1)(p22),add(8),del(16)	Adverse	Neg	Neg	BM	83.4
12	79.30	Diagnosis	Normal	Intermediate	ND	ND	BM	65.1
13	79.98	Diagnosis	44,XX,−3,−5,add(5)(p15) or del(5)(p13),inv(7)(p22q11.2),+i(11)(q10),add(12)(p13),−17[cp20]	Adverse	ND	ND	BM	58.7
14	48.48	Refractory	Normal	Intermediate	Neg	ND	PB	89.4
15	37.99	Refractory	Normal	Intermediate	Neg	Pos	PB	92.5
16	23.92	Diagnosis	46,XX,inv(16)(p13q22)(17]/47,sdl,+22[3]	Favorable	Neg	Neg	PB	92.2
17	68.71	Diagnosis	Normal	Intermediate	Pos	Pos	PB	93.5
18	73.77	Diagnosis	91,XXXX,−5,del(6)(q21q25)[20]	Adverse	Neg	Neg	BM	86.5
19	36.16	Relapse	47,XY,+8[20]	Intermediate	Pos	Neg	BM	85.8
20	54.43	Diagnosis	46,XY,inv(16)(p13.1q22)[20]	Favorable	Neg	Neg	PB	92.5
21	64.67	Diagnosis	91,XXYY,del(5)(q23q31),del(6)(q15q23),−7,del(7)(q31)[10]/94~99,sl,+6,+8,+9,+10,+13,+14,+19,+19,+20,+22,+0~1mar[cp6]/46,XY[4]	Adverse	Pos	Neg	BM	38.6
22	66.51	Diagnosis	Normal	Intermediate	Pos	Pos	BM	93.2
23	51.43	Diagnosis	Normal	Intermediate	Pos	Pos	PB	86.1
24	69.10	Refractory	Normal	Intermediate	Pos	Pos	PB	87.1
25	52.13	Diagnosis	Normal	Intermediate	Neg	Neg	PB	70.2
26	40.16	Diagnosis	Normal	Intermediate	Pos	Pos	PB	75.2
27	40.56	Relapse	Normal	Intermediate	Pos	Pos	PB	90.3
28	55.69	Refractory	Normal	Intermediate	Pos	ND	PB	83.2
29	48.30	Refractory	46,XY,t(11;12)(q13;p13)[9]/46,XY[1]	Intermediate	Neg	Neg	PB	87.4

Abbreviations: BM, bone marrow; ND, not done; neg, negative; PB, peripheral blood; pos, positive.

### Generation of cell line models to study CD33 splice variants

**Figure 3:**
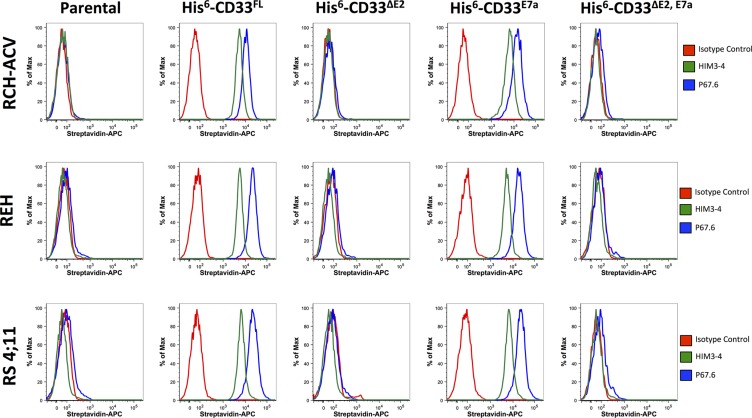
Phenotyping of acute leukemia cell lines with CD33 antibodies Parental RCH-ACV, REH, and RS4;11 cells and their sublines transduced with His-tagged CD33^FL^, CD33^∆E2^, CD33^E7a^, or CD33^∆E2,E7a^ were incubated with HIM3-4, P67.6 or isotype control antibody followed by incubation with a biotin-anti-mouse secondary antibody and APC-streptavidin. Results are shown from one representative experiment.

**Figure 4:**
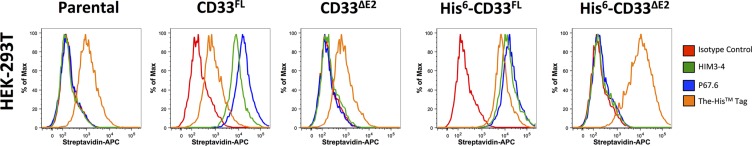
Phenotyping of engineered HEK293T cells with CD33 antibodies Parental HEK293T cells and sublines transduced with untagged or His-tagged CD33^FL^ or CD33^∆E2^ were incubated with HIM3-4, P67.6, a His antibody, or isotype control antibody followed by incubation with a biotin-anti-mouse secondary antibody and APC-streptavidin. Results are shown from one representative experiment.

**Figure 5:**
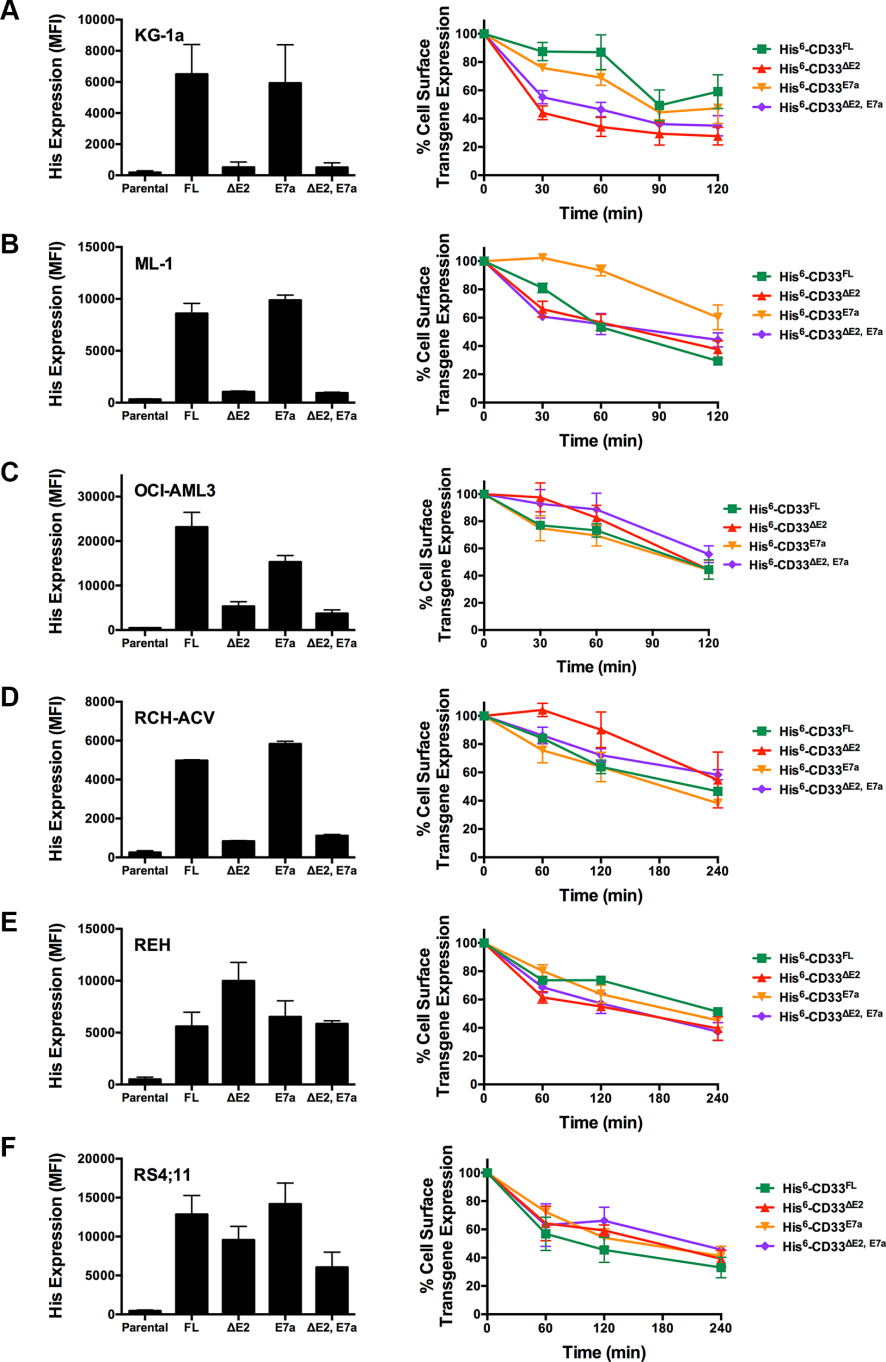
Internalization of CD33 splice variants in engineered acute leukemia cell lines Parental (**A**) KG-1a, (**B**) ML-1, (**C**) OCI-AML3, (**D**) RCH-ACV, (**E**) REH, and (**F**) RS4;11 cells were transduced with lentiviral particles to express either His-tagged CD33^FL^, CD33^∆E2^, CD33^E7a^, or CD33^∆E2,E7a^. *LEFT PANEL*: Cell surface display of the tagged transgenes was then quantified flow cyometrically using His antibody, and results expressed as arbitrary median fluorescence units (MFI). *RIGHT PANEL:* Internalization of His antibody was quantified in aliquots of engineered leukemia cell sublines over 120 or 240 minutes, and shown as percentage of remaining cell surface expression of the CD33 transgene detected at time 0. Results are depicted as mean ± SEM from 3 independent experiments.

Having demonstrated the presence of CD33^∆E2^, CD33^E7a^, and CD33^∆E2,E7a^ mRNA in primary human AML cells, we then studied the functional characteristics of encoded proteins. Therefore, we generated cell line models of human acute leukemia in which we selectively expressed untagged or His-tagged CD33^FL^, CD33^∆E2^, CD33^E7a^ or CD33^∆E2,E7a^ via lentivirus-mediated gene transfer, using a series of human AML (KG-1a, ML- 1, and OCI-AML3) and acute lymphoblastic leukemia (ALL; RCH-ACV, REH, and RS4;11) cell lines. We used tagged versions of the CD33 proteins to distinguish those from endogenously expressed CD33 in some of these cell lines and to be able to track the CD33^∆E2^ and CD33^∆E2,E7a^ variants. While previous studies suggested that one CD33 antibody (clone Him3-4) specifically recognizes the C_2_- set Ig-like domain and could be used to detect CD33 variants that lack exon 2 [9], in our experiments Him3- 4 only recognized CD33^FL^ and CD33^E7a^ but not CD33^∆E2^ or CD33^∆E2,E7a^when expressed in virally-transduced ALL cell lines that are devoid of endogenous CD33 (Jurkat, RCH-ACV, REH, or RS4;11: Figure 3) and only recognized CD33^FL^ but not CD33^∆E2^ when expressed in virally-transduced HEK293T cells (Figure 4). As shown in Figure 5, engineered sublines expressed high amounts of CD33^FL^ and CD33^E7a^ in all cell lines. Lentivirus-mediated gene transfer also resulted in readily detectable cell surface display of the CD33^∆E2^ and CD33^∆E2,E7a^ splice variants, although the level of expression varied considerably across cell line backgrounds.

### Internalization of CD33 splice variants in human leukemia cell lines

**Figure 6:**
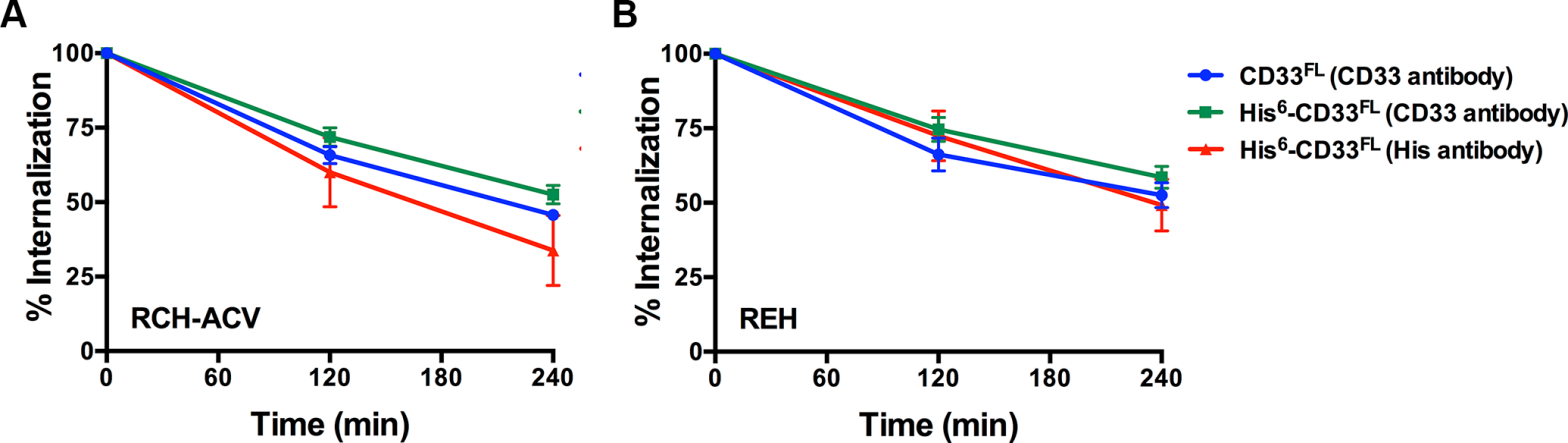
Internalization of antibody-bound CD33 Comparative analysis of the 120-minute and 240-minute internalization of untagged and His-tagged CD33^FL^ when bound with CD33 antibody or His antibody in lentivirally-transduced (**A**) REC-ACV and (**B**) REH cells. Results are shown as mean ± SEM from 3 independent experiments.

**Figure 7:**
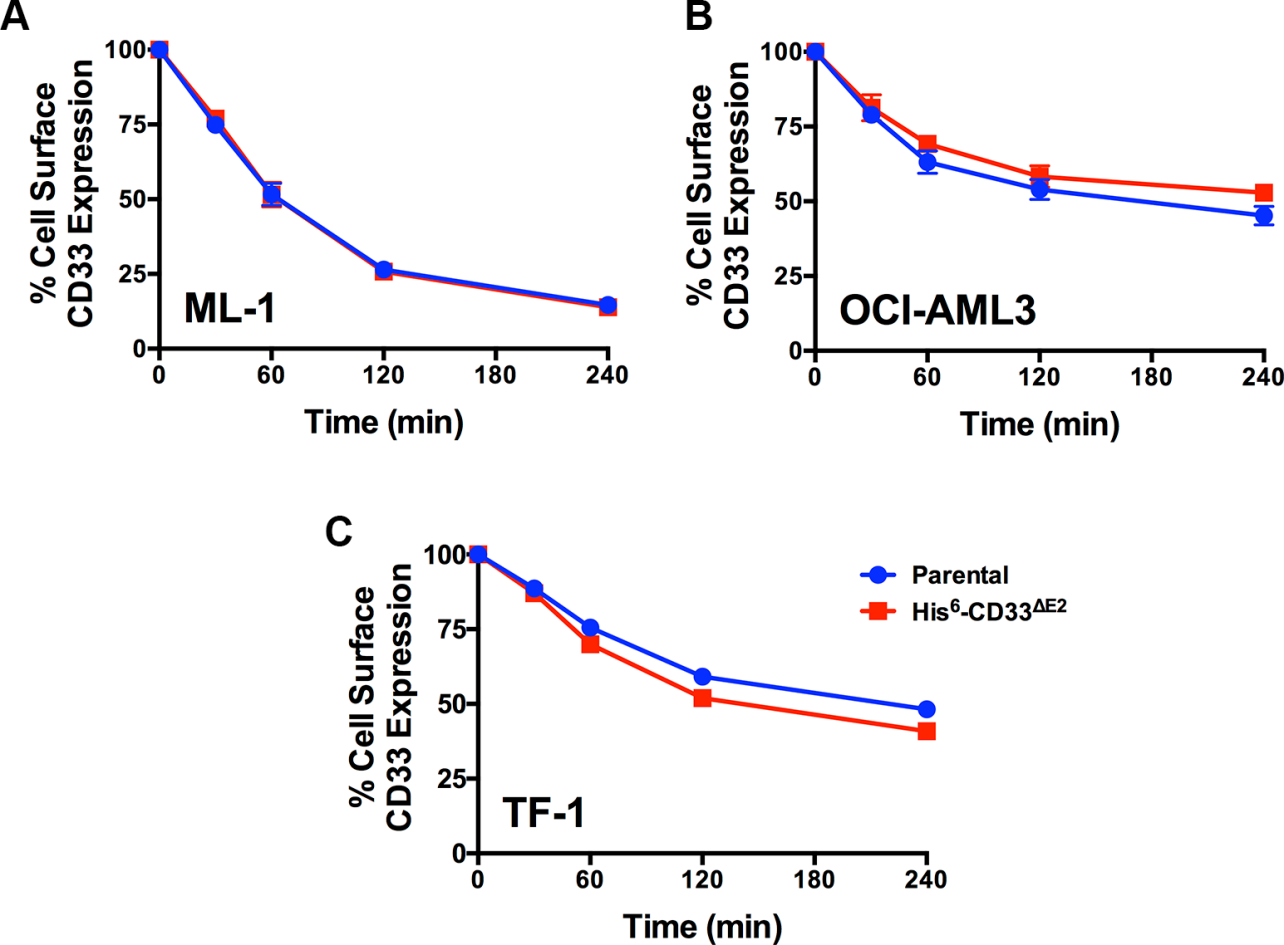
Effect of CD33^∆E2^ on internalization of CD33^FL^ Parental (**A**) ML-1, (**B**) OCI-AML3, and (**C**) TF-1 cells and their sublines transduced with His-tagged CD33^∆E2^ were incubated with CD33 antibody, and internalization of antibody-bound CD33 was then quantified flow cyometrically over 240 minutes and shown as percentage of remaining cell surface expression of CD33 detected at time 0. Results are depicted as mean ± SEM from 3 independent experiments.

**Figure 8:**
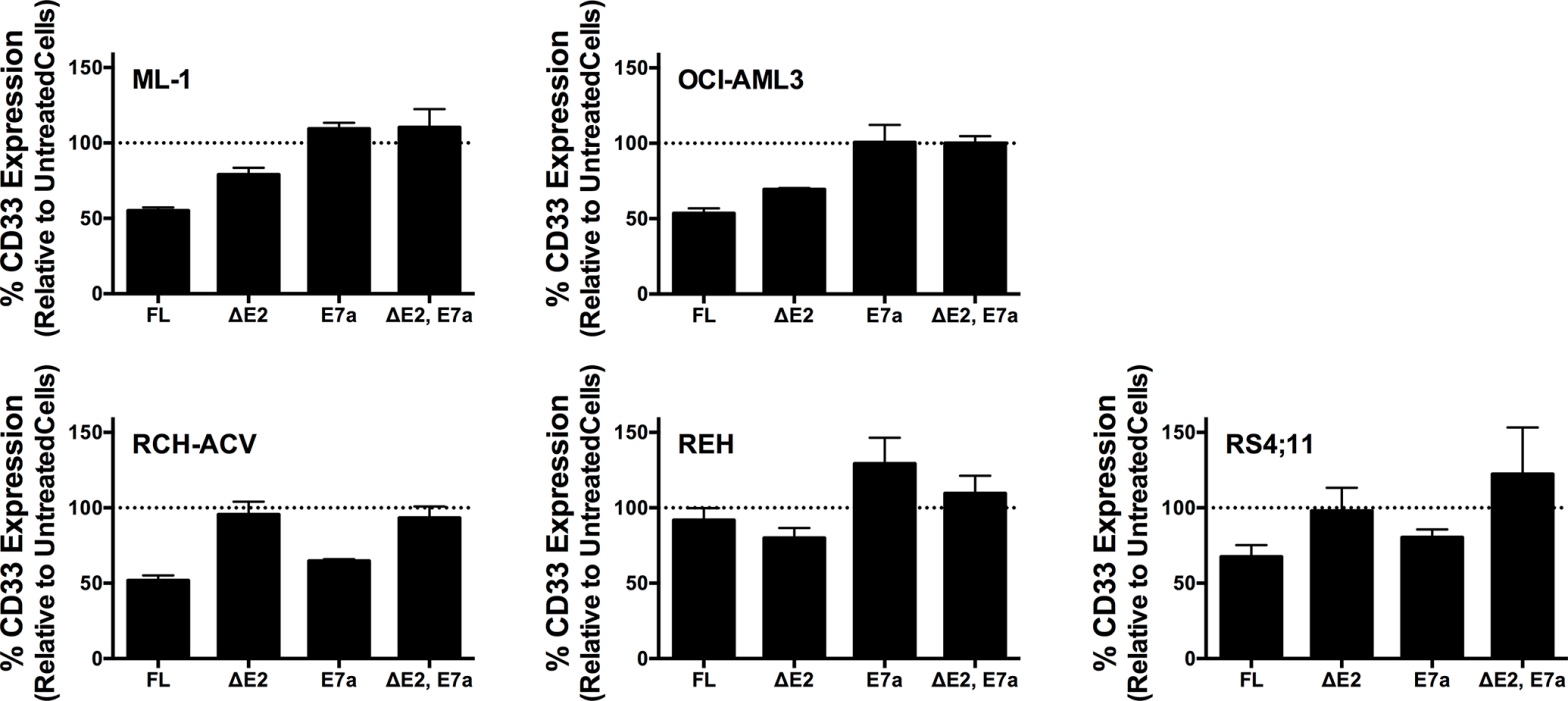
CD33 modulation in engineered acute leukemia cells Comparative analysis of the modulation of His-tagged CD33^FL^, CD33^∆E2^, CD33^E7a^, or CD33^∆E2,E7a^ in lentivirally-transduced AML cells (ML-1, OCI-AML3) and ALL cells (RCH-ACV, REH, RS4;11). Cells were incubated for 24 hours with His antibody followed by incubation with a biotin-anti-mouse secondary antibody and APC-streptavidin. Results are shown as mean ± SEM from 3 independent experiments.

As the first characteristic, we studied the endocytic properties of the CD33 splice variants when engaged by bivalent antibodies. To test whether the internalization of antibody-bound CD33 differed depending on the type of antibody used (i.e. His antibody vs. CD33 antibody), we compared the 120- and 240-minute internalization of CD33 in RCH-ACV and REH engineered to expressed untagged and His-tagged CD33^FL^. The studies showed very comparable rates of antibody uptake when studied with CD33 antibody in cells expressing untagged and His-tagged CD33^FL^ or with His antibody in cells expressing His-tagged CD33^FL^, indicating that the His-tag does not interfere with the natural internalization properties of CD33 (Figure 6). Overall, our experiments in the panel of sublines of AML and ALL cells expressing His-tagged CD33 spice variants demonstrated that each variant is internalized when bound by a bivalent His antibody (Figure 5). While, on average, the internalization properties of each variant appeared relatively similar, we noted some cell line-specific differences in the comparative uptake of individual CD33 splice variants. In particular, in ML-1 cells, internalization of the CD33^E7a^ variant proceeded more slowly than that of the other CD33 molecules. Since some previous data have suggested that CD33 might form dimers on the cell surface in its physiologic state [16], we considered the possibility that CD33^∆E2^ could interfere with the functional properties of CD33^FL^. To test this idea, we compared the internalization of endogenous CD33 when bound with CD33 antibody in ML-1, OCI-AML3, and TF-1 cells that were lentivirally-transduced to also express CD33^∆E2^ with that of non-transduced parental cells. These studies showed that uptake of endogenous CD33 was unaffected by the forced expression of the CD33^∆E2^ splice variant (Figure 7). Finally, we also assessed the degree to which cell surface CD33 expression levels decreased with continued exposure to antibody (“CD33 modulation”). In 24-hour assays, we found some differences between individual CD33 variants in several acute leukemia cell lines; however, while highly reproducible, these differences were not consistent across cell lines but, rather, appeared cell context specific. Specifically, in engineered AML cell lines, a reduced degree of antigen modulation relative to wild-type CD33 was noted in sublines expressing CD33 isoforms containing exon 7a; by comparison, in engineered ALL cell lines, a reduced degree of antigen modulation relative to wild-type CD33 was noted in sublines expressing CD33 isoforms that lack exon 2 (Figure 8).

### GO-induced cytotoxicity in human leukemia cell lines expressing CD33 splice variants

**Figure 9:**
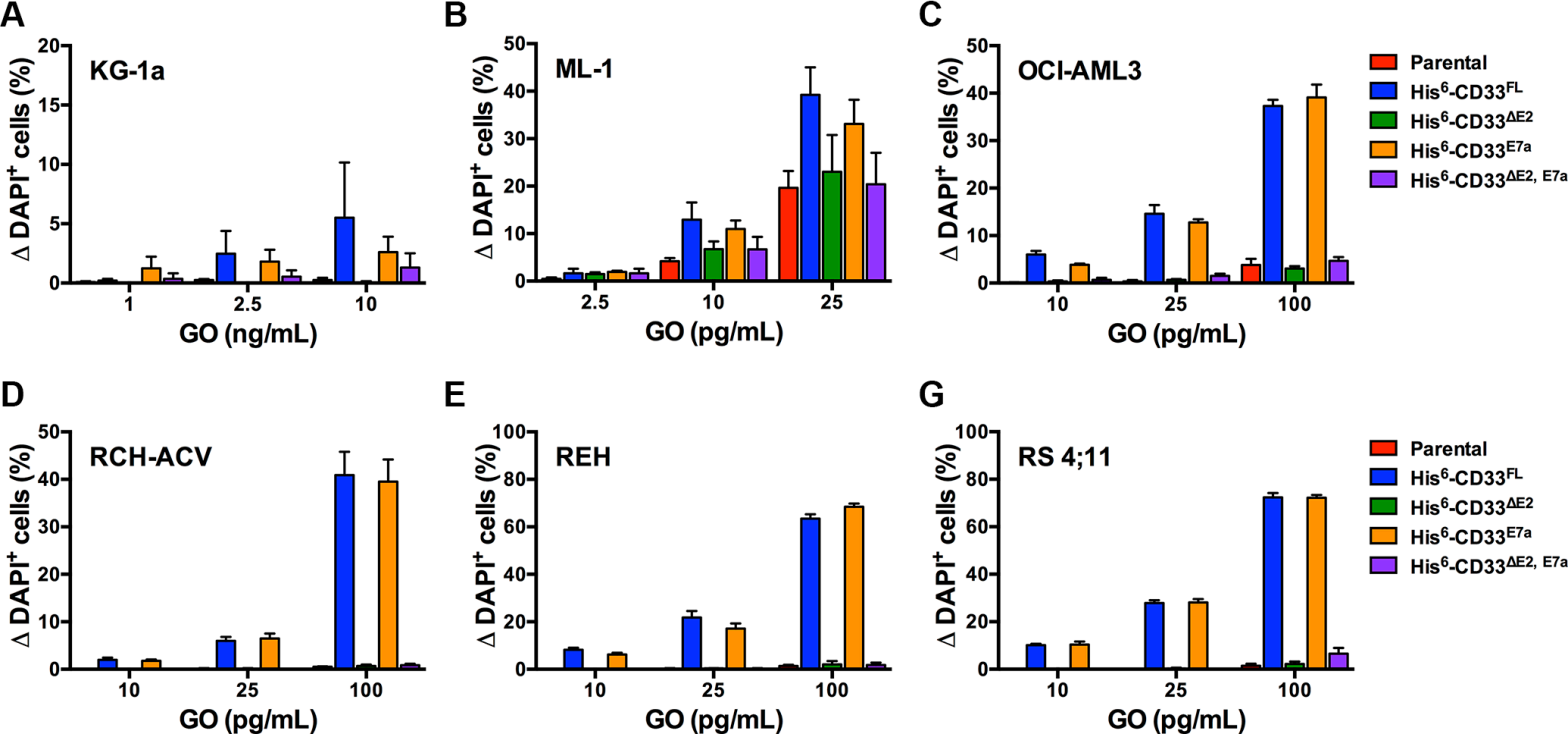
Effect of CD33 splice variants on GO-induced cytotoxicity Parental (**A**) KG-1a, (**B**) ML-1, (**C**) OCI-AML3, (**D**) RCH-ACV, (**E**) REH, and (**F**) RS4;11 cells and their sublines transduced with His-tagged CD33^FL^, CD33^∆E2^, CD33^E7a^, or CD33^∆E2,E7a^ were then incubated with various concentrations of GO for 3 days before cytotoxicity was assessed with DAPI staining. Increases in the percentage of DAPI^+^ cells in GO-treated cells are compared to corresponding cells that were incubated without GO, and results are shown as mean ± SEM from 3 independent experiments performed in duplicate wells.

As the second characteristic, we studied the cytotoxic properties of the CD33 antibody-drug conjugate GO in parental AML and ALL cell lines and corresponding sublines engineered to express His-tagged CD33 splice variants. As shown in Figure 9, expression of CD33 splice variants that contain the V-set domain recognized by GO (CD33^FL^ and CD33^E7a^) sensitized human AML and ALL cell lines to GO-induced cytotoxicity. Consistent with our previous data [13], overexpression of CD33^FL^ increased GO-induced cytotoxicity in AML cell lines (KG-1a, ML-1, and OCI-AML3) that endogenously express CD33. In contrast, expression of CD33 variants that do not contain the V-set domain (CD33^∆E2^ and CD33^∆E2,E7a^) did not modulate the cytotoxic effects of GO. Moreover, co-expression of CD33^∆E2^ did not impact GO-induced cytotoxicity in AML cells endogenously displaying CD33 on the cell surface, as evidenced by our findings in ML-1 and OCI-AML3 cells, indicating that CD33^∆E2^ does not interfere with the ability of CD33^FL^ to serve as carrier for intracellular delivery of cytotoxic drugs to CD33-expressing leukemia cells.

## DISCUSSION

The number of antigens exploited for immunotherapy of AML has been increasing rapidly over the last several years [17]. Nonetheless, CD33 remains of great interest for the development of antibody-based therapies. Besides its broad expression in AML, this interest is at least partially due to two circumstances, namely the fact that it thus far represents the only validated target in AML and the fact that GO – the antibody-drug conjugate that provided target validation – is currently not commercially available in most countries [1]. Several new CD33-directed agents, including a second-generation antibody-drug conjugate (SGN- CD33A), a bispecific T-cell engaging (BiTE) antibody (AMG 330), and chimeric antigen receptor-modified T-cells have recently entered the clinical phase of drug testing, and others are at various stages of preclinical development.

Because of this ongoing interest in CD33 as an AML target, we sought to better characterize the expression pattern of various forms of CD33 in human AML. Using a large set of diagnostic specimens from patients with AML, our studies demonstrate that in the vast majority of cases, 3 splice variants of CD33 are found in addition to CD33^FL^. One of these variants (CD33^∆E2^) has previously been found in normal myeloid cells, microglial cells, T-lymphocytes, and natural killer (NK) cells [8–11, 18], and has been implicated in influencing the susceptibility to Alzheimer's disease [10, 11, 18]. CD33^∆E2^ has also been described in several human AML cell lines [9]. We extend these findings by demonstrating that CD33^∆E2^ is present at the mRNA level in the vast majority of primary AML specimens (29 of 29 in our series). Our studies also show the almost universal presence of mRNA corresponding to a CD33^E7a^ splice variant (the sequence of which was previously deposited in the genome databases; found in 29 of 29 in our series) and a CD33^∆E2,E7a^ splice variant (found in 27 of 29 in our series), which has so far not been described.

Exon 2 encodes the ligand-binding extracellular V-set domain of CD33, and CD33 proteins lacking this exon are expected to feature functional properties that differ from those of wild-type CD33. Indeed, previous studies have indicated that CD33^FL^ but not CD33^∆E2^ inhibits uptake and clearance of insoluble, toxic amyloid β species by microglial cells *in vitro* – an activity that may be important for the pathogenesis of Alzheimer's disease [19]. These studies have identified CD33 as a potential target for the treatment and/or prevention of Alzheimer's disease [19]. In this situation, blocking CD33 isoforms that contain exon 2 and have functional activity may be sufficient. For the treatment of CD33+ malignancies, however, directing the therapeutic toward all CD33 isoforms displayed on the cell surface may be advantageous since it would provide the greatest possible abundance of antibody binding sites. Consistent with previous reports [9, 19], we found that CD33 variants lacking exon 2 can be expressed on the cell surface, although our studies indicate that the efficiency with which this happens varies between different cell line backgrounds. Similar to wild-type CD33, our experiments in engineered acute leukemia cell lines document that the CD33^∆E2^ variant is internalized, and could thus also serve as target for CD33-directed therapeutics that depend on intracellular delivery of a toxic payload.

Our studies in lentivirally-transduced acute leukemia cell lines also show that the CD33 variants that contain exon 7a, and therefore lack almost the entire cytoplasmic tail of CD33, are internalized. At first glance, this is surprising since we previously found CD33 endocytosis to be controlled by the intracellular domain of CD33. Introduction of point mutations in this domain, for example in the immunoreceptor tyrosine-based inhibitory motifs or clusters of lysine residues, reduced internalization of antibody/CD33 complexes in our earlier studies [13, 15]. Whether the cell membrane localization is influenced by the presence of the cytoplasmic tail of CD33, and whether the mechanistic principles for the uptake process differ between wild-type CD33 and variants that contain exon 7a, is currently unknown and will be subject of future investigations. The cell context-specific differences we found with regard to modulation of individual CD33 variants would be consistent with such differences in membrane localization and/or internalization mechanisms. Planned studies will also aim to identify differences between CD33 variants containing exon 7a with wild-type protein with regard to suppression of myeloid cell function – we hypothesize that such differences exist given the lack of immunoreceptor tyrosine-based inhibitory motifs when exon 7a is utilized.

In summary, our studies demonstrate the presence of 3 splice variants of CD33 that have endocytic properties when bound by a bivalent antibody in almost all patients with AML. These findings document a greater-than-previously thought complexity of CD33 expression in human AML. Furthermore, they identify CD33 variants that lack exon 2 and are not recognized by currently explored CD33-directed therapeutics as potential, hitherto unexploited, targets for immunotherapy with unconjugated or conjugated CD33 antibodies.

## MATERIALS AND METHODS

### Transcriptome sequencing of primary AML specimens

Sixty-eight patients with newly diagnosed AML enrolled on 2 recent COG trials were selected for retrospective whole transcriptome RNA sequencing (RNAseq) because they lacked known high-risk cytogenetic features but eventually relapsed [20]. Total RNA from pre-treatment bone marrow or peripheral blood specimens was used to generate a cDNA library, which was purified and enriched by polymerase chain reaction (PCR) amplification and subjected to 50-cycle paired-end sequencing on the Illumina HiSeq as previously described [20]. RNA sequencing reads were then aligned to the human genome to identify splice junctions between exons and identify new gene variants using TopHat [21]. Informed consent was obtained in accordance with the Declaration of Helsinki. The institutional review boards (IRBs) of all participating institutions approved the clinical protocol, while the Fred Hutchinson Cancer Research Center (Fred Hutch) IRB and the COG Myeloid Disease Biology Committee approved this research study.

### PCR-based detection of CD33 splice variant expression in primary AML specimens

Frozen aliquots of Ficoll-isolated mononuclear cells from pretreatment specimens (bone marrow [*n* = 13], peripheral blood [*n* = 14], or apheresis [*n* = 2]) from 29 adults with newly diagnosed (*n* = 17) or relapsed/refractory (*n* = 12) AML were obtained from Fred Hutch AML cell repositories and selected for inclusion based on the presence of a large number of myeloblasts (median: 87.4%; range: 38.6–95.1%; Table 1). We used the refined United Kingdom Medical Research Council/National Cancer Research Institute (MRC/NCRI) criteria to assign cytogenetic risk [22]. Patients provided written informed consent for the collection and use of their specimens for research purposes under an IRB-approved protocol. Genetic material was extracted using AllPrep DNA/RNA Mini Kits (Qiagen, Valencia, CA). Approximately 25 ng of RNA was then reverse transcribed for each PCR reaction with specific primers to amplify CD33^FL^, CD33^∆E2^, CD33^E7a^, and CD33^∆E2,E7a^ (see Table 2 for primer sequences). Amplicons were electrophoretically separated in 0.9% agarose gel and visualized with ethidium bromide (AlphaImager HP; ProteinSimple, San Jose, CA, USA). Selected amplicons were subjected to Applied Biosystems (ABI) BigDye™ Terminator sequencing.

**Table 2:** List of primers to detect CD33 splice variants

CD33 variant	Primer sequences
CD33^FL^	For: 5′-TGGCTATGGATCCAAATTTCTGGCTG-3′ (annealing to exon 2) Rev: 5′-AGCATAATGCAGCTCCTCATC-3′ (annealing to exon 7b)
CD33^∆E2^	For: 5′-CCTGCTGTGGGCAGACTTGAC-3′ (annealing to junction of exon 1/exon 3) Rev: 5′-AGCATAATGCAGCTCCTCATC-3′ (annealing to exon 7b)
CD33^E7a^	For: 5′-TGGCTATGGATCCAAATTTCTGGCTG-3′ (annealing to exon 2) Rev: 5′-GATTGCAGGTGTGAAACACTG-3′(annealing to exon 7a)
CD33^∆E2,E7a^	For: 5′-CCTGCTGTGGGCAGACTTGAC-3′ (annealing to junction of exon 1/exon 3) Rev: 5′-GATTGCAGGTGTGAAACACTG-3′(annealing to exon 7a)

### Generation of lentiviral vectors expressing CD33 constructs

A pRRLsin.cPPT.MSCV lentivirus containing a human CD33^FL^ in conjunction with an internal ribosomal entry site (IRES)/Enhanced Green Fluorescent Protein (EGFP) cassette has previously been described [13, 23]. Human cDNAs corresponding to CD33^FL^ with an N-terminal hexahistidine (His), and the CD33^∆E2^, CD33^E7a^, and CD33^∆E2,E7a^ variants with or without an N-terminal His tag were generated in TOPO vectors via standard PCR cloning procedures (see Table 3 for primer sequences), verified by sequencing, and subsequently transferred into the pRRLsin.cPPT.MSCV vector. Lentiviral particles were prepared as described previously [13].

**Table 3:** List of mutagenesis primers

Construct	Primer sequences
CD33^FL/His^	For: 5′-CATCATCACCATCACCACGGAGGTGGAATGGATCCAAATTTCTGGCTGC-3′ Rev: 5′-TCCACCTCCGTGGTGATGGTGATGATGAGCCAGGGCCCCTGC-3′
CD33^∆E2^	For: 5′-TGCCCCTGCTGTGGGCAGACTTGACCCACAGGCCC-3′ Rev: 5′-GGGCCTGTGGGTCAAGTCTGCCCACAGCAGGGGCA-3′
CD33^∆E2/His^	For: 5′-CATCATCACCATCACCACGGAGGTGGACCCAAAATCCTCATCCCTG-3′ Rev: 5′-TCCACCTCCGTGGTGATGGTGATGATGCCTGTGGGTCAAGTCTGC-3′
CD33^E7a^, CD33^E7a/His^, CD33^∆E2,E7a^, CD33^∆E2,E7a/His^	For: 5′-ATGCCGCTGCTGCTACTGCTGC-3′ Rev: 5′-TCAACGTACCGGGGAGGCTGACCCTGTGGTAG-3′

### Parental and engineered human cell lines

Human myeloid KG-1a cells (kindly provided by Dr. Derek L. Stirewalt; Fred Hutch, Seattle, WA), OCI-AML3 cells (kindly provided by Dr. Soheil Meshinchi; Fred Hutch), and ML-1 as well as TF-1 cells (kindly provided by Dr. Irwin D. Bernstein; Fred Hutch) were maintained as previously described [13, 24, 25]. Human lymphoid RCH-ACV and REH cells (kindly provided by Dr. Jerald P. Radich; Fred Hutch) were maintained in RPMI-1640 medium (Life Technologies, Grand Island, NY) with 10% fetal bovine serum (FBS; HyClone, Thermo Scientific, Logan, UT), and RS4;11 cells were maintained in MEM Alpha (Life Technologies) with 10% FBS. Human embryo kidney (HEK)293T cells (kindly provided by Dr. Jonathan A. Cooper; Fred Hutch) were maintained in DMEM medium (Life Technologies) with 10% FBS. All cell lines were confirmed to be mycoplasma-free but were not recently authenticated. Sublines of these cell lines overexpressing individual tagged or untagged CD33 constructs were generated through transduction with the appropriate pRRLsin.cPPT.MSCV lentiviral particles at a multiplicity of infection (MOI) of 25. EGFP-positive cells were isolated by flow cytometry and re-cultured for further analysis.

### Quantification of CD33 expression

Expression of untagged CD33 variants on leukemia cell lines was quantified by flow cytometry using an unconjugated CD33 antibody (clone P67.6; BD Biosciences, San Jose, CA, USA) [13, 23] or unconjugated CD33 antibody followed by a biotin-anti-mouse secondary antibody and APC-streptavidin (both BD Biosciences). In some cases, a second CD33 antibody (clone HIM3-4) previously reported to recognize the C2-set Ig-like domain of CD33 [9] was used to detect cell surface display of CD33 variants. Expression of hexahistidine-tagged CD33 proteins was determined using an antibody against THE™ His tag (clone 6G2A9; GenScript, Piscataway, NJ, USA) followed by a biotin-anti-mouse secondary antibody and APC-streptavidin. To identify nonviable cells, samples were stained with 4′,6-diamidino-2-phenylindole (DAPI). 10,000 events were acquired on a BD FACSCanto II flow cytometer (BD Biosciences), and DAPI^−^ cells analyzed using FlowJo (Tree Star, Ashland, OR).

### Quantification of CD33 internalization and modulation

To measure internalization of antibody-bound CD33, cells (typically 1–1.5 × 10^6^) were transferred into 15 mL polypropylene conical bottom tubes (Falcon™, Corning Life Sciences, Tewksbury, MA, USA) and incubated for at least 20 minutes with phosphate buffered saline (PBS, GIBCO by Life Technologies)/2% FBS containing 2.5 μg/mL unconjugated P67.6 (Santa Cruz Biotechnology, Dallas, TX, USA) or His tag antibody in ice-water (to prevent internalization during the staining procedure), as appropriate. Cells were then washed in ice-cold PBS, resuspended in IMDM medium without antibody, split into several tubes, and incubated at 37°C (in 5% CO_2_ and air) for various periods of time. Afterwards, cells were chilled and incubated with biotin-conjugated rat anti-mouse IgG_1_ monoclonal antibody (used at 2.5 μg/mL in PBS/2% FBS), followed by incubation with streptavidin-APC conjugate (used at 2.5 μg/mL in PBS/2% FBS: both from BD Biosciences, San Jose, CA, USA) to detect remaining primary antibody on the cell surface. One sample that was kept in ice water was used to determine the starting level of antibody bound to the cell surface. To determine CD33 modulation, aliquots of engineered leukemia cells were left untreated or incubated with unlabeled His tag antibody (used at 2.5 μg/mL). After 24 hours, cells were washed in ice-cold PBS to remove unbound antibody and resuspended in PBS/2%FBS. Aliquots of untreated and antibody-treated cells were then incubated with His antibody (to saturate binding sites) or no antibody followed by biotin-conjugated rat anti-mouse IgG_1_ (used at 2.5 μg/mL in PBS/2% FBS) and streptavidin-APC (used at 2.5 μg/mL in PBS/2% FBS). For analysis, all samples were stained with DAPI to identify nonviable cells; at least 10,000 events were acquired, and DAPI^−^ cells were analyzed on a BD FACSCanto II flow cytometer using FlowJo. Linear fluorescence values were used to calculate the percentage of CD33 internalization or modulation.

### Quantification of drug-induced cytotoxicity

AML cells were incubated at 37°C (in 5% CO_2_ and air) in 96-well round bottom plates (Falcon™) at 8 × 10^3^ cells/well in 200 μL culture medium containing various concentrations of gemtuzumab ozogamicin (commercially obtained from Pfizer, New York, NY). After 72 hours, cell numbers and drug-induced cytotoxicity, using DAPI to detect non-viable cells, were determined using a BDFACSCanto II flow cytometer and analyzed with FlowJo software.
